# A bioengineered anti‐VEGF protein with high affinity and high concentration for intravitreal treatment of wet age‐related macular degeneration

**DOI:** 10.1002/btm2.10632

**Published:** 2023-12-19

**Authors:** Chengnan Huang, Yuelin Wang, Jinliang Huang, Huiqin Liu, Zhidong Chen, Yang Jiang, Youxin Chen, Feng Qian

**Affiliations:** ^1^ School of Pharmaceutical Sciences, Beijing Frontier Research Center for Biological Structure, and Key Laboratory of Bioorganic Phosphorus Chemistry & Chemical Biology (Ministry of Education) Tsinghua University Beijing People's Republic of China; ^2^ Department of Ophthalmology Peking Union Medical College Hospital, Peking Union Medical College, Chinese Academy of Medical Sciences Beijing People's Republic of China; ^3^ Key Lab of Ocular Fundus Diseases, Chinese Academy of Medical Sciences Beijing People's Republic of China; ^4^ Quaerite Biopharm Research Beijing People's Republic of China; ^5^ Present address: Department of Anesthesia University of California at San Francisco San Francisco California USA

**Keywords:** high concentration protein, intravitreal injection, multivalency, nanobody, protein engineering

## Abstract

Intravitreal (IVT) injection of anti‐vascular endothelial growth factor (anti‐VEGF) has greatly improved the treatment of many retinal disorders, including wet age‐related macular degeneration (wAMD), which is the third leading cause of blindness. However, frequent injections can be difficult for patients and may lead to various risks such as elevated intraocular pressure, infection, and retinal detachment. To address this issue, researchers have found that IVT injection of anti‐VEGF proteins at their maximally viable concentration and dose can be an effective strategy. However, the intrinsic protein structure can limit the maximum concentration due to stability and solution viscosity. To overcome this challenge, we developed a novel anti‐VEGF protein called nanoFc by fusing anti‐VEGF nanobodies with a crystallizable fragment (Fc). NanoFc has demonstrated high binding affinity to VEGF_165_ through multivalency and potent bioactivity in various bioassays. Furthermore, nanoFc maintains satisfactory chemical and physical stability at 4°C over 1 month and is easily injectable at concentrations up to 200 mg/mL due to its unique architecture that yields a smaller shape factor. The design of nanoFc offers a bioengineering strategy to ensure both strong anti‐VEGF binding affinity and high protein concentration, with the goal of reducing the frequency of IV injections.

**Translational Impact Statement**

Our team has developed an innovative anti‐VEGF protein called nanoFc, which could enhance the treatment for retinal disorders, specifically wet age‐related macular degeneration (wAMD), a major cause of blindness. NanoFc exhibits high binding affinity to VEGF_165_, potent bioactivity, and satisfactory stability. Its unique architecture allows for concentrations up to 200 mg/mL, reducing the need for frequent intravitreal injections. This bioengineering advancement has the potential to significantly improve patient quality of life and decrease risks associated with wAMD treatment.

## 1 INTRODUCTION

Intravitreal (IVT) injection of anti‐VEGF (vascular endothelial growth factor) proteins is a commonly used treatment for various retinal conditions, particularly those involving abnormal blood vessel growth, such as wet age‐related macular degeneration (wAMD), diabetic macular edema (DME), retinal vein occlusion (RVO), retinopathy of prematurity (ROP), and myopic choroidal neovascularization (mCNV). By targeting VEGF, a protein that plays a crucial role in the formation of abnormal blood vessels in the retina, these medications help reduce the growth of new blood vessels and prevent leakage from existing blood vessels, thereby preserving vision and preventing further damage.^1^, ^2^, ^3^ For example, after anti‐VEGF therapies for wAMD, 90%–95% of patients clinically show a positive response.^4^, ^5^

The FDA approved the first anti‐VEGF drug, Pegaptanib, in 2004,^6^, ^7^ after a long journey involving various therapies such as antioxidant, photodynamic and radiation therapy, as well as transpupillary thermotherapy, which revolutionized the treatment of wAMD.^8^ Over the next 20 years, anti‐VEGF biologics such as ranibizumab, bevacizumab (off‐label use), aflibercept, conbercept, brolucizumab and faricimab were subsequently approved and became the standard care for wAMD, with intervals of intravitreal injection ranging from approximately 1–4 months.^9^, ^10^, ^11^ However, frequent IVT injections can be difficult for patients due to fear or discomfort, lack of understanding, lack of healthcare and financial support, forgetfulness or complexity, personal beliefs or preferences, etc.^12^, ^13^, ^14^, ^15^ Frequent IVT injections can also potentially raise risks for other problems or complications, such as vein damage, infection, phlebitis, thrombosis, extravasation, fluid and electrolyte imbalances, allergic reactions.^16^

Recently, the development of high‐affinity and high concentration proteins has been demonstrated to be an effective and practical strategy for prolonging the duration of anti‐VEGF action and reducing the frequency of injections. Enhancing the binding affinity and increasing the molar amounts of anti‐VEGF proteins can both promote the neutralization of VEGF.^17^, ^18^, ^19^ For example, aflibercept, a first‐line drug for wAMD, was further developed to a high concentration of 115 mg/mL. Clinical trial data have demonstrated that an 8 mg dose of highly‐concentrated aflibercept extends the injection interval from 2 months to 4 months while meeting the clinical endpoints of non‐inferiority.^20^, ^21^ This achievement potentially positions the 8 mg dose aflibercept as a best‐in‐class biotherapeutic product for wAMD.

However, pursuing both high affinity and high concentration can pose a challenge as they can be inherently contradictory goals when applying conventional affinity maturation approaches.^22^ Typically, rounds of site mutations are employed to improve binding affinity, either through display technology or guided by computational analysis, to alter non‐covalent interactions such as hydrogen bonds, electrostatic force, van der Waals force, and hydrophobic interactions between antigen and complementary‐determining region (CDR) of the antibody. However, manipulating protein–protein interactions, especially those involving charge and hydrophobic interactions, carries inherent risks when developing a high concentration formulation.^23^, ^24^

In addition to affinity maturation through rounds of site mutations, high binding affinity can also be achieved by utilizing multivalent proteins connected with optimal linkers. The basic principle has been demonstrated in a previous work.^25^ In this study, our objective was to develop a high‐affinity and high concentration protein through protein design rather than formulation optimization. To this end, we created an Fc‐fused tetravalent nanobody (nanoFc) using the multivalency strategy to enhance affinity and evaluated the feasibility of this novel molecule for developing a high concentration formulation. NanoFc exhibited satisfactory colloidal and conformational stability and good anti‐VEGF potency without rounds of site mutations or the introduction of undesirable charge or hydrophobic amino acids. Additionally, the architecture and surface properties of nanoFc also contributed to its high concentration and low viscosity characteristics.

## 2 MATERIALS AND METHODS

### 2.1 Expression and purification of recombinant proteins

The Technology Center for Protein Research at Tsinghua University provided the HEK293‐F cell line and the pVRC8400_tPA plasmid, while the pCDNA3.1 plasmid was purchased from Invitrogen (Thermo Fisher Scientific, USA). To express N1H (a model anti‐VEGF nanobody obtained from a Patent^26^), N2H‐9GS (a bivalent nanobody with two N1H nanobody connected by a GGGSGGGGS linker), we cloned them into the pVRC8400_tPA plasmid, while the pCDNA3.1 plasmid was used to clone the Fc‐fused multivalent nanobody (nanoFc). HEK293‐F cells were cultured in SMM 293‐TII medium (Sinobiological, China) at 5% CO_2_ and 37°C for 4–5 days. The cells were transfected with the plasmid and PEI (Polysciences, USA) at a final concentration of 1 μg/mL and 3 μg/mL, respectively, at a density of 1.5–2.0 × 10^6^ cells/mL after being sub‐cultured for 3–4 passages. N1H and N2H‐9GS were purified using Ni beads and protein A beads (Smart Life sciences, China) were used to purify nanoFc in the supernatant. The purity of the protein was evaluated using SDS‐PAGE or size‐exclusion high‐performance liquid chromatography (SEC‐HPLC), while the concentration was measured by UV absorbance at 280 nm (Nanodrop 2000, Thermo Scientific, Wilmington, DE, USA). The concentration analyzer Lunatic (Unchained Labs, USA) was employed to determine the concentration of nanoFc when it was over 50 mg/mL.

### 2.2 Determination of isoelectric point (pI)

The pI of nanoFc was determined using Capillary Isoelectric Focusing (cIEF, CESI 8000 plus, AB Sciex, Sweden). To accomplish this, 10 μL of nanoFc (5 mg/mL) was mixed with 200 μL Urea‐cIEF gel (3 M, Sigma–Aldrich, USA), 12 μL ampholytes (3–10, Sigma–Aldrich, USA), 20 μL arginine (500 mM, Sigma–Aldrich, USA), 20 μL iminodiacetic acid (200 mM, Sigma–Aldrich, USA), and 2 μL pI marker (Sigma–Aldrich, USA), and subsequently loaded onto the system. The mixture was prefocused for 15 min at 25 kV, followed by a chemical mobilization at 30 kV for 30 min. The whole‐column UV detector at 280 nm was used to acquire the main component and charge variants after they were focused on their respective pI values.

### 2.3 Surface plasmon resonance (SPR)

The binding affinity of nanoFc to VEGF was determined using surface plasmon resonance (SPR) on a Biacore S200 instrument (GE, USA). VEGF_165_ (GenScript, Nanjing, China) was immobilized onto flow channels of an activated CM5 sensor‐chip by diluting it to 5 μg/mL in 10 mM sodium acetate (pH 5.5) and injecting into the SPR system to obtain a final immobilization level of 160 RU. A range of nanoFc analyte dilutions were subsequently injected, with HBS‐EP running buffer (GE‐Healthcare, USA) at a flow rate of 45 μL/min for 150 s, followed by a dissociation time of 1200 s. The sensor chip surface was regenerated with 100 mM HCl (Merck, China) between each injection. The binding sensorgrams were analyzed using the 1:1 Langmuir model to determine the binding kinetics and dissociation constant.

### 2.4 Enzyme linked immunosorbent assay (ELISA)

ELISA was used to determine the EC50 binding ability of recombinant proteins to VEGF_165_ and to quantify the concentration of anti‐VEGF proteins in samples from the aqueous chamber, vitreous humor and ocular tissue. Briefly, a 96‐well plate was coated overnight with 100 μL of 300 ng/mL VEGF_165_ (GenScript, Nanjing, China), followed by washing with 150 μL PBST three times, and immobilization with 5% non‐fat milk solution (Solarbio, Beijing, China) for 1 h at room temperature. Diluted proteins were added to the wells and incubated for 1 h at room temperature. Subsequently, 100 μL of antibodies (His‐tag antibody HRP‐66005, Proteintech, Wuhan, China, for N1H and N2H‐9GS; HRP, Goat Anti‐Human IgG, Abbkine, Wuhan, China, for nanoFc) were added to the plates and incubated for 1 h at room temperature to capture recombinant proteins. Lastly, 100 μL of TMB substrate solution (Tiangen, Beijing, China) and 50 μL of stop solution (Solarbio, Beijing, China) were sequentially added to the wells. The results were read at OD450 using a microplate reader (SpectraMax Gemini XPS/EM Microplate Readers, Molecular Devices, USA).

### 2.5 HUVEC proliferation assay

The anti‐VEGF ability and resulting inhibition efficiency of cell growth for the recombinant proteins and control were assessed using the HUVEC proliferation assay. In brief, a series of 4‐fold dilutions starting from 750 nM were prepared using 0.5% FBS MEM (Zhong Qiao Xin Zhou Biotech., Shanghai, China) for the test protein. Next, 50 μL of 210 ng/mL VEGF_165_ (GenScript, Nanjing, China) in 0.5% FBS MEM was added to each dilution of the test protein in a 96‐well plate (Costar, Corning, Inc. USA), followed by incubation at 37°C for 1.5–2 h. Then, HUVEC cells (NSCTRB, Shanghai, China) were prepared at a concentration of 2.4 × 10^5^ cells/mL, and 50 μL of the cell suspension was added to each well. After incubation for 68–72 h, 16 μL of CCK‐8 (Solarbio, Beijing, China) was added to each well of the plate, followed by incubation at 37°C for 2.5 h. The absorbance at 450 nm was measured using a microplate reader (Multiskan™ Spectrophotometer Thermo Scientific, Singapore).

### 2.6 Reporter gene assay

The anti‐VEGF efficiency of recombinant proteins was compared using a HEK293 reporter gene‐based assay, which has been previously described.^27^ Briefly, HEK293 cells were transfected with VEGFR2 and NFAT‐RE‐luc2p genes, and activation of the NFAT transcription factor, which occurs upon triggering of the VEGF‐VEGFR2 pathway, promotes the expression of the Luciferase gene, which can be detected using luciferin substrate. The cells were sub‐cultured in 1% FBS DMEM (Gibico, Thermo Fisher Scientific, USA) at a density of 5 × 10^5^ cells/mL and were added to a 96‐well white plate (Costar, Corning, Inc., USA) at a volume of 80 μL to incubate overnight at 37°C. The test protein was serially diluted three times from 100 nM using 1% FBS DMEM, and the same volume (60 μL) of 80 ng/mL VEGF_165_ in 1% FBS DMEM was added to each dilution. The mixtures were incubated at 37°C for 30 min, and then 20 μL of the mixture was added to the cells for 6 h of incubation at 37°C until luminescence measurement (Firefly Glo Luciferase Reporter Gene Assay Kit, YEASEN, Shanghai, China).

### 2.7 Phosphorylation measurement of VEGFR2

The binding of VEGF_165_ to VEGFR2 triggers phosphorylation of VEGFR2, which is typically located at Tyr1175.^28^ Therefore, the bioactivity of test proteins can be determined by detecting the phosphorylation level through western blot analysis. HUVEC cells were sub‐cultured with 5% FBS MEM to a density of 3 × 10^5^ cells/mL, and a volume of 100 μL was added to a 96‐well plate for overnight incubation at 37°C. Three dilutions of the test protein (0.92 nM, 2.80 nM and 8.40 nM) were made by 5% FBS MEM, and the same volume (75 μL) of 100 ng/mL VEGF_165_ in 5% FBS MEM was added to each dilution. The mixtures in 96‐well plate were subsequently incubated at 37°C for 30 min. Cells were treated by 4.5 μL of 36 mM Na_3_VO_4_ (Sigma–Aldrich, USA) for 5 min at 37°C. Then, 100 μL of the mixtures were added to the treated cells for 5 min of incubation. Cells were later lysed with 65 μL of RIPA (Beyotime Biotechnology, Shanghai, China), followed by shaking at 4°C for 20 min and centrifugation for harvesting the supernatant. The concentration of total protein was determined and adjusted before western blot analysis.

Western blot analysis was performed as follows: 20 μL of each sample were loaded onto a 4%–12% SDS‐PAGE gel and transferred onto a PVDF membrane. The membrane was blocked with 5% non‐fat milk in TBST buffer (20 mM Tris–HCl, 150 mM NaCl, 0.05% Tween‐20, pH 7.6) for 1 h at room temperature and then incubated with primary antibody against phosphorylated VEGFR2 (pTyr1175) (Cell Signaling Technology, USA) at 1:2000 dilution in TBST buffer overnight at 4°C. The membrane was then washed with TBST buffer and incubated with secondary antibody (HRP‐conjugated goat anti‐rabbit IgG, Beyotime Biotechnology, Shanghai, China) at 1:8000 dilution in TBST buffer for 1 h at room temperature. After washing with TBST buffer, the membrane was developed using an ECL kit (Beyotime Biotechnology, Shanghai, China) and exposed to x‐ray film.

### 2.8 Analysis of protein size, unfolding temperature (*T*_m_) and aggregation temperature (*T*_agg_)

UNcle (Unchained Labs, CA, USA) was used to determine the size, unfolding temperature (*T*_m_) and aggregation temperature (*T*_agg_) of the proteins (N1H, N2H‐9GS and nanoFc). The physical stability of these samples was assessed using *T*_m_ and *T*_agg_. Prior to analysis, the samples were diluted to a concentration of 1 mg/mL in PBS and then loaded onto the sample track using a 9 μL volume. Each sample was measured three times to ensure accuracy.

### 2.9 Size‐exclusion chromatography (SEC)

The detection of nanoFc aggregates during formulation studies was performed using a Prominence‐i9 high‐performance liquid chromatography system (LC‐2030, Shimadzu, Japan) coupled with Zenix‐C SEC‐300 (3 μm, 7.8 × 300 mm, Sepax Technologies, DE, USA). Each sample, containing 1 mg/mL of nanoFc, was loaded onto the system in a volume of 50 μL and eluted at 30°C with a mobile phase consisting of 150 mM sodium phosphate at pH 6.8 and a flow rate of 0.6 mL/min.

### 2.10 Weak cation exchange chromatography (WCX)

Prominence‐i9 high‐performance liquid chromatography system (LC‐2030, Shimadzu, Japan) coupled with WCX column (BioCore WCX, NanoChrom, China) was utilized to determine the charge heterogeneity of nanoFc during formulation studies. The following chemicals and parameters were used: running buffer A consisted of 20 mM PBS (pH 6.5), running buffer B was composed of 20 mM PBS and 1000 mM NaCl (pH 6.5), flow rate was set to 0.5 mL/min, and the temperature was maintained at 40°C.

### 2.11 Prediction and analysis of protein structure

The prediction of protein structure was accomplished through the utilization of AlphaFold 2, which was constructed in‐house in accordance with the instructions provided on the following repository: https://github.com/deepmind/AlphaFold. Surface properties of proteins were subsequently analyzed using BioLuminate (Schrodinger Inc., NY, USA). The shape factor was calculated using formula as follows:

$$F=\frac{s}{\sqrt[{3}]{v^{2}}}$$

where *F*, *s*, and *v* represent shape factor, surface area, and volume, respectively.

### 2.12 Determination of solution viscosity

To determine the viscosity profile of the protein solution, MicroVISC (Rheosense Inc., USA) coupled with a Type A chipset (14HA05100550) was utilized. The detailed methodology has been described elsewhere.^24^ Prior to each measurement, water was utilized as a reference liquid to ensure the accuracy of the rheometer. The average viscosity values, based on 3–5 measurements, were subsequently recorded.

### 2.13 In vivo pharmacokinetics

The PK study was conducted using rabbits that were purchased from Beijing Fangyuan Farm and were handled in accordance with the guidelines set by the Institutional Animal Care and Use Committee (IACUC). The study involved the preparation of nanoFc at a concentration of 3.3 mg/mL to meet the dose requirement of 0.1 mg per injection of 30 μL. The samples were stored at 4°C before use. A Hamilton trace syringe and 30‐gauge needle were used for intravitreal injection. Animals were given 0.2 mL of Xylazine Hydrochloride for anesthesia through intraperitoneal injection, and ocular sanitization was done by Tropicamide Phenylephrine Eye Drops before administering the anti‐VEGF proteins.

Three rabbits were used for each time point for each test sample, while only 5 eyes were injected with proteins, and the left was given the same volume of buffer to determine the interaction between the two eyes in a single rabbit. Sampling was performed at 1 h, day 1, day 2, day 5, day 9, day 14, and day 30. Aqueous humor, vitreous humor, and tissue (We defined the remaining components of the enucleated eye, after removing the aqueous and vitreous humor, as tissue. This tissue included the ciliary body, choroid/retinal pigment epithelium, retina, iris, sclera, and bulbar conjunctiva) were collected and stored at −20°C. All samples were pretreated before ELISA analysis, such as centrifugation at 12,000 rpm for 10 min to collect supernatant for aqueous and vitreous humor, and lysing the tissue with 1 mL PBS (with protease inhibitor) via homogenizer, following with 12,000 rpm centrifugation for 10 min to collect supernatant.

ELISA was used to determine the protein concentration of each sample. A standard curve was established in each plate, and the diluted sample concentrations fell within the standard curve. PKSolver was used to analyze the PK parameters according to the dependence of protein concentration on time.^29^

### 2.14 Data analysis

Statistical analyses were performed using GraphPad Prism Software (San Diego, CA, USA), and the mean ± standard deviation (SD) was used to present the data. To determine significance, a two‐tailed unpaired Student's *t*‐test was utilized. Different degrees of significance were considered (ns *p* > 0.05, *p < 0.05, **p < 0.01, ***p < 0.001, ****p < 0.0001).

## 3 RESULTS AND DISCUSSION

### 3.1 Rational design and expression of recombinant proteins for IVT injection

**FIGURE 1:**
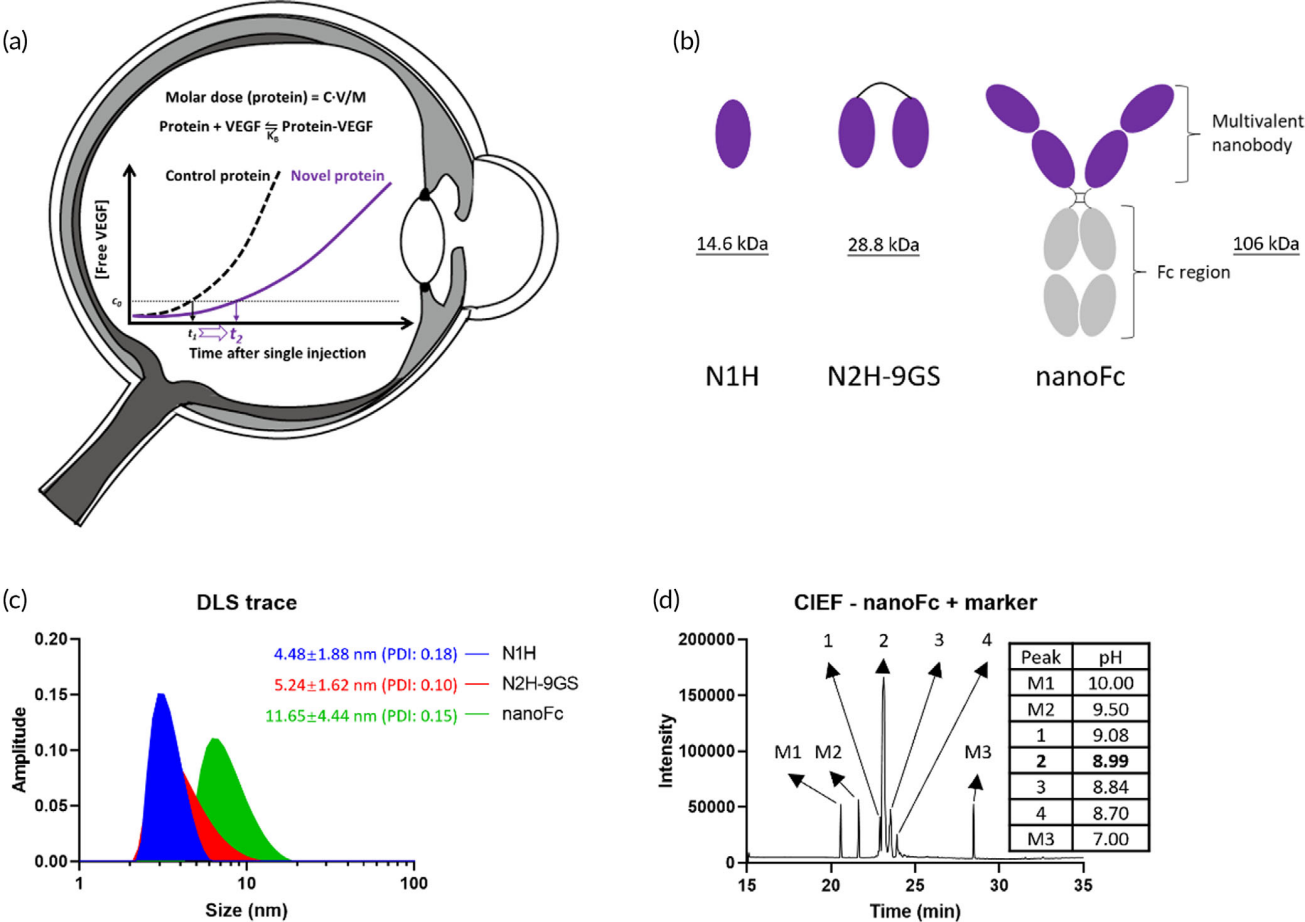
Design and characterization of anti‐VEGF proteins. (a) Binding equilibrium of protein‐VEGF in the vitreous chamber. Parameters *c*, *v*, *M*, *K*_*B*_ represent concentration, injection volume, molecular weight, binding constant, respectively. A higher concentration and lower molecular weight lead to a higher molar dose of protein, which, together with binding affinity, promotes the interaction between anti‐VEGF protein and VEGF, thereby prolonging the duration of VEGF neutralization; (b) schematic representation of N1H (a monovalent anti‐VEGF nanobody), N2H‐9GS (a bivalent nanobody, with two N1H nanobodies connected by a GGGSGGGGS linker) and nanoFc (Fc‐fused tetravalent nanobodies); (c) DLS profiles of N1H, N2H‐9GS, and nanoFc; (d) determination of isoelectric point for nanoFc by CIEF. Peaks M1–M3 indicate marker, while peaks 1–4 correspond to nanoFc.

The treatment of wet age‐related macular degeneration (wAMD) involves intravitreal (IVT) injection of anti‐VEGF proteins. However, frequent IVT injections can be difficult for patients due to various reasons. Next‐generation protein drugs designed for intravitreal (IVT) injection, targeting VEGF_165_, offer a potential solution. A novel protein for IVT injection must meet requirements such as achieving high molar dose, affinity, bioactivity, solubility and stability. The objective of this study was to develop a high‐affinity and high concentration (or high molar dose) protein for intravitreal (IVT) injection, with both properties promoting the binding reaction between the protein and VEGF, thereby extending the duration of VEGF inhibition (Figure 1a).

Achieving a higher molar dose for IVT injection can be accomplished by lowering the molecular weight, as discussed above, and by developing a high concentration formulation. However, it has been challenging to obtain a molecule with both high affinity and high concentration. One reason for this difficulty is that the affinity‐optimized CDR region typically contains strong hydrophobic and electrostatic interactions, which can hinder the development of high concentration formulations.^30^ It is evident that developing a high‐affinity and high concentration protein is inherently contradictory if the conventional affinity maturation approach is applied.

To address this challenge, herein we constructed an Fc‐fused tetravalent nanobody (nanoFc, Figure 1b) that consists of four nanobodies and an Fc region with a molecular weight of 106 kDa. Bivalent nanobodies connected by a flexible GS linker were fused directly to the *N*‐terminal of a single chain of the IgG1 Fc region, and intact nanoFc was formed through the formation of a disulfide bond at the hinge region of Fc. NanoFc was harvested with a yield of ~200 mg/mL through mammalian cell cultivation and protein A purification processes, ~20 times higher than that of bivalent nanobodies N2H‐9GS, which presumably was contributed by the existence of Fc region in the fusion protein.^31^ NanoFc was expected to be feasible for high affinity and high concentration because multivalency, as a strategy to construct high‐affinity proteins, can eliminate the introduction of extra hydrophobic or charged amino acids that are typically associated with the conventional maturation process. Furthermore, the addition of the Fc region was expected to physically stabilize the protein, and contributed to the development of high concentration formulation.^31^

To further characterize the molecular properties of nanoFc, we measured its size, purity, pI, and charge variants, which are critical quality attributes for the development of high concentration formulations. As shown in Figure 1c, nanoFc has a hydrodynamic size of 11.65 ± 4.44 nm and a small polydispersity index (PDI), indicating a very high size purity without aggregates and fragments produced during the entire process due to misfolding or chemical degradation. The CIEF profile presented the charge variants and pI of nanoFc (Figure 1d). Two acidic peaks (peak 3 and 4) and one basic peak (peak 1) were considered degradation products of the main peak (peak 2, pI = 8.99). Possible degradation pathways could include *N*‐glycosylation, deamidation, isomerization, *C*‐terminal clipping, and *N*‐terminal blocking.^32^

### 3.2 VEGF binding affinity and in vitro bioactivity

**FIGURE 2:**
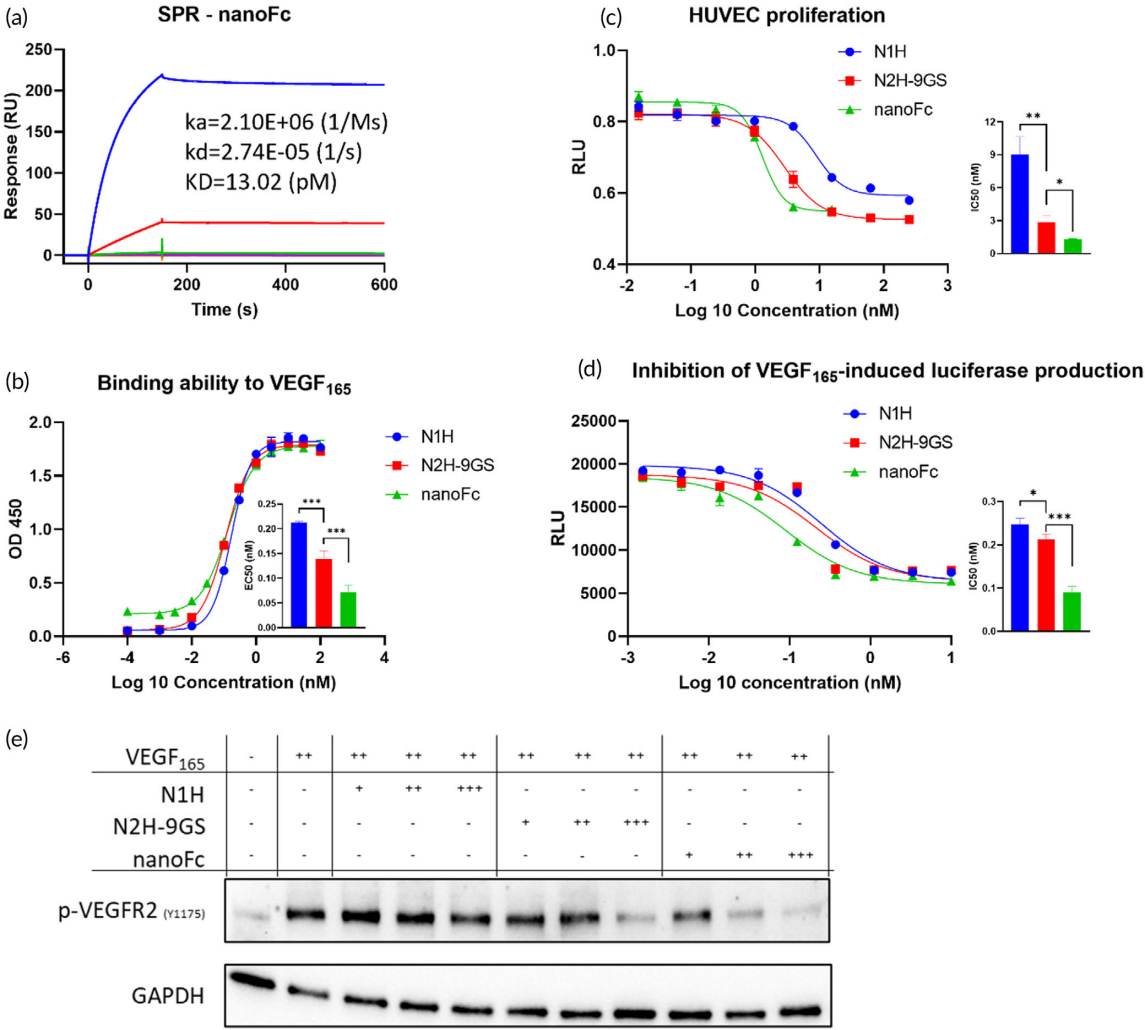
Characterization of binding affinity and bioactivity of anti‐VEGF proteins in vitro. (a) Binding kinetics of nanoFc as determined by SPR; (b) ELISA profile and EC50 of N1H, N2H‐9GS, and nanoFc to VEGF_165_; (c) inhibition of N1H, N2H‐9GS, and nanoFc on HUVEC proliferation; (d) inhibition of N1H, N2H‐9GS, and nanoFc on VEGF_165_‐induced luciferase production; (e) inhibition of N1H, N2H‐9GS, and nanoFc on the phosphorylation of VEGFR2, which is triggered by VEGF_165_, as determined by western blot. “−” “+” “++” “+++” represent a concentration of 0 nM, 0.46 nM, 1.40 nM, and 4.20 nM, respectively.

Potent binding affinity with VEGF and in vitro bioactivity of novel proteins are critically important for their potential therapeutic applications. In this study, we evaluated the binding affinity of nanoFc to VEGF_165_ using SPR and ELISA, and the cell level bioactivity using three cell‐based assays: HUVEC proliferation, reporter gene, and phosphorylation. SPR is widely recognized as the gold standard for capturing antibody–antigen interactions. The fusion protein, nanoFc, exhibited improved binding affinity (13.02 pM, Figure 2a) when compared to those of N1H and N2H‐9GS as reported previously,^25^ in which we found that connecting two nanobodies by a 9GS linker increased the binding affinity of the bivalent nanobody. ELISA results supported this finding, with EC50 values of 0.21 nM, 0.14 nM, and 0.07 nM for N1H, N2H‐9GS, and nanoFc, respectively (Figure 2b). These results demonstrated that our strategy to construct (Fc‐fused) multivalent nanobodies was effective in improving the binding affinity to pM range.

To evaluate the bioactivity of the novel proteins, we performed three cell‐based assays. HUVEC proliferation assay showed that all anti‐VEGF proteins inhibited cell growth in a dose‐dependent manner, with IC50 values of 9.04 nM, 2.86 nM, and 1.29 nM for N1H, N2H‐9GS, and nanoFc, respectively (Figure 2c). Reporter gene assay showed that all the proteins inhibited the VEGF_165_‐induced luciferase production, with IC50 values of 0.25 nM, 0.21 nM, and 0.09 nM for N1H, N2H‐9GS, and nanoFc, respectively (Figure 2d). Western blot analysis revealed that the phosphorylation signal of VEGFR2 was inhibited in a dose‐dependent manner by all the proteins tested. The inhibition trend was more significant when we increased the valency of nanobody from mono to tetravalent (Figure 2e).

Taken together, the multivalent nanobodies (i.e., N1H, N2H‐9GS, and nanoFc) showed increased VEGF binding affinity and bioactivity with the increase of valency, confirming the feasibility of the multivalency strategy to meet the requirement of biological activity of VEGF neutralization without multiple rounds of conventional affinity maturation procedures.

### 3.3 Physical stability and feasibility of high concentration protein formulation

**FIGURE 3:**
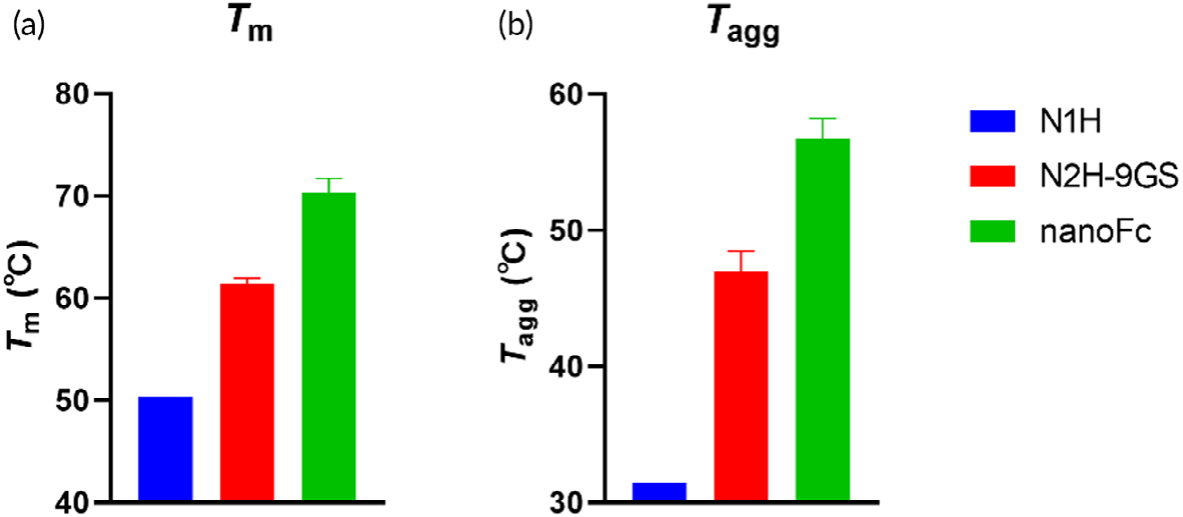
Protein physical stability comparison of N1H, N2H‐9GS, and nanoFc as determined by UNcle. (a) Unfolding temperature; (b) aggregation temperature. The measurements were performed in triplicate.

After optimizing the binding affinity, our next objective was to develop a high concentration formulation for the well‐designed proteins. During preliminary testing, we observed that the bivalent nanobody N2H‐9GS was unstable and prone to aggregation at concentrations above 10 mg/mL, which was supported by the physical stability characterization data (Figure 3). N1H and N2H‐9GS exhibited low unfolding and aggregation temperatures (*T*_m_ and *T*_agg_). In contrast, nanoFc had a *T*_m_ of 70.4°C and a *T*_agg_ of 56.7°C, suggesting potentially good conformational and colloidal stability. Therefore, together with the high yield of nanoFc expression, we selected nanoFc for further investigation of high concentration formulation development.

**FIGURE 4:**
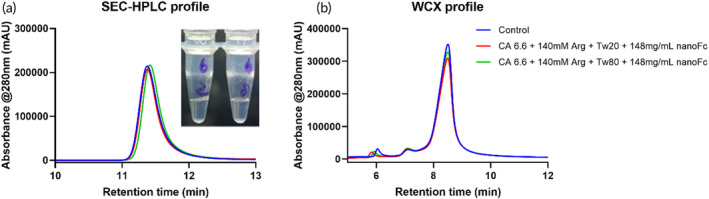
SEC (a) and WCX (b) characterization of highly‐concentrated nanoFc after 28 days of storage at 4°C. The insert in (a) shows the appearance of solutions containing 148 mg/mL nanoFc following formulation #6, which consists of 20 mM citric acid‐sodium citrate buffer (pH 6.6), 140 mM arginine·HCl, and Tween‐20 or −80 (2 and 8 on the tube, respectively, represent Tween‐20 and Tween‐80).

Solution properties of protein, including their surface charge and protein–protein interaction vary significantly in different solution environments, leading to differences in net interaction energy or energy barrier in the DLVO balance, which could subsequently impact protein colloidal stability.^33^ After analyzing 18 different buffer systems (Figure S1), 20 mM pH 5.0 acetate acid‐sodium acetate buffer (AC 5.0), 20 mM pH 5.6 acetate acid‐sodium acetate buffer (AC 5.6), and 20 mM pH 6.6 citrate acid‐sodium citrate buffer (CA 6.6) appeared to be the more suitable systems based on stability analysis. Furthermore, viscosity reducers or stabilizers, including 140 mM NaCl, 140 mM arginine·HCl, or 40 mM NaCl in combination with 5% sucrose, were tested for the development of high concentration nanoFc. Among the eight conditions tested (Table S1), the formulations with 140 mM arginine·HCl were able to reach a high nanoFc concentration. Based on more stability comparison under 4°C or 25°C of selected formulations (Table S2), formulation #6 outperformed other formulations in terms of stability (Figures S2 and S3). SEC‐HPLC (Figure 4a) demonstrated 100% purity and recovery of nanoFc after a 28‐day stability study, while the WCX results (Figure 4b) showed that the main peak accounted for 92.96%, 92.00%, and 91.67% in the control, Tween‐20 formulation, and Tween‐80 formulation, respectively. These data collectively indicate good chemical and colloidal stability for nanoFc in formulation #6, suggesting promising potential for future development.

### 3.4 Viscosity‐concentration relationship of nanoFc: role of protein architecture and surface properties

**FIGURE 5:**
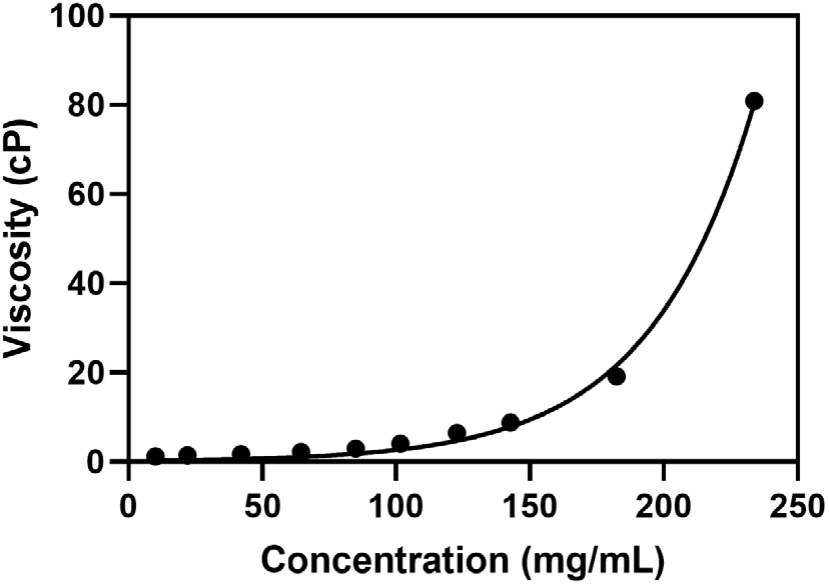
Dependence of viscosity (measured at 25°C) on concentration for nanoFc in the formulation: 20 mM citrate acid—sodium citrate buffer, pH 6.6, 140 mM arginine·HCl. The measurements and analyses were conducted with three replicates and error bars were included.

Moreover, we profiled the dependence of viscosity on concentration for nanoFc at 25°C (Figure 5), which represents its ease in the purification process and developability in formulating into high concentration. It appears that the novel protein nanoFc had a good viscosity performance. For example, the viscosity of nanoFc at a concentration of 120 mg/mL was only 6 cP, which was lower than that of 8 mg dose aflibercept (around 15 cP at 115 mg/mL),^34^ an recently approved formulation for wAMD treatment with longer injection intervals.^20^, ^21^ Furthermore, nanoFc possessed injectable viscosity at a concentration even up to 200 mg/mL (In‐house testing using a 29G needle).

**FIGURE 6:**
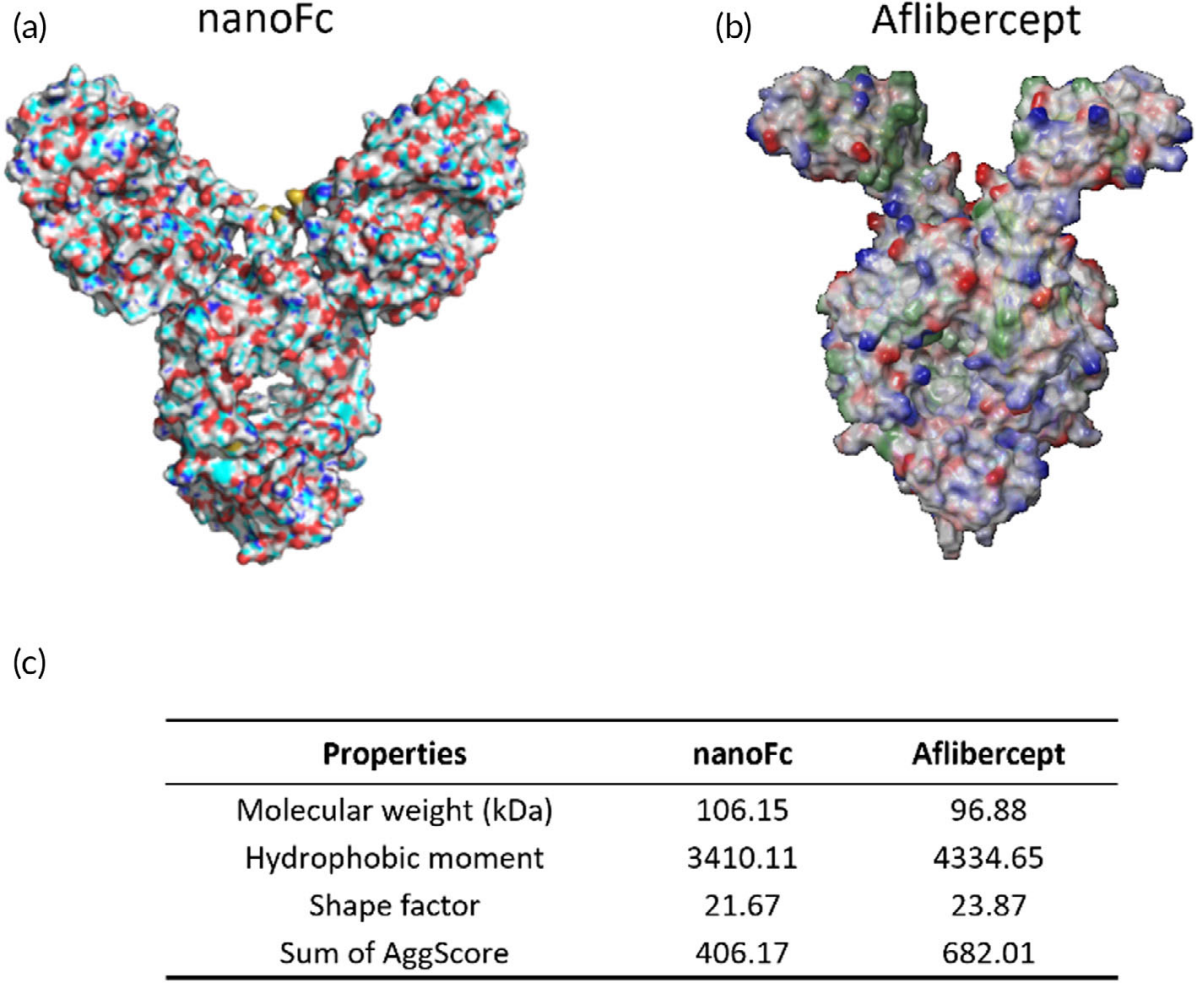
Architecture and surface analyses of nanoFc. (a, b) Structures of nanoFc and aflibercept, which were predicted by AlphaFold 2; (c) table of properties related to protein stability and viscosity. The molecular weight was calculated based solely on the amino acids, without taking into account glycosylation or any other post‐translational modifications. The hydrophobic moment and sum of AggScore of the two proteins were analyzed using BioLuminate 3.4.

Next, we attempted to analyze the fusion protein nanoFc from a structural perspective to reveal its performance in terms of viscosity and stability. First, we obtained the three‐dimensional structures of the fusion protein nanoFc and aflibercept through AlphaFold 2 (as shown in Figure 6a,b). To further compare the differences in the distribution of hydrophobic patches between the two proteins, we analyzed the surfaces of both proteins using BioLuminate 3.4. We found that nanoFc exhibited an overall smaller hydrophobic area, approximately 22% less than aflibercept (Figure 6c), suggesting that nanoFc had a weaker hydrophobic effect compared to aflibercept. By comparing the shape factors of the two proteins, we observed that the architecture of the fusion protein nanoFc was more spherical. This resulted in nanoFc having lower intrinsic viscosity and thus demonstrating better viscosity performance. Furthermore, by analyzing the AggScore of both protein types,^35^ we discovered that nanoFc had a lower predicted value than aflibercept, indicating that nanoFc had a lower tendency to aggregate and higher stability.

Through structural analysis, we found that nanoFc had a more spherical three‐dimensional structure. Additionally, its distribution of surface hydrophobic regions and aggregation tendency were significantly lower than those of aflibercept. These properties collectively contributed to the advantages of nanoFc in formulation development, such as low viscosity and high stability.^36^, ^37^, ^38^

### 3.5 In vivo pharmacokinetics

**FIGURE 7:**
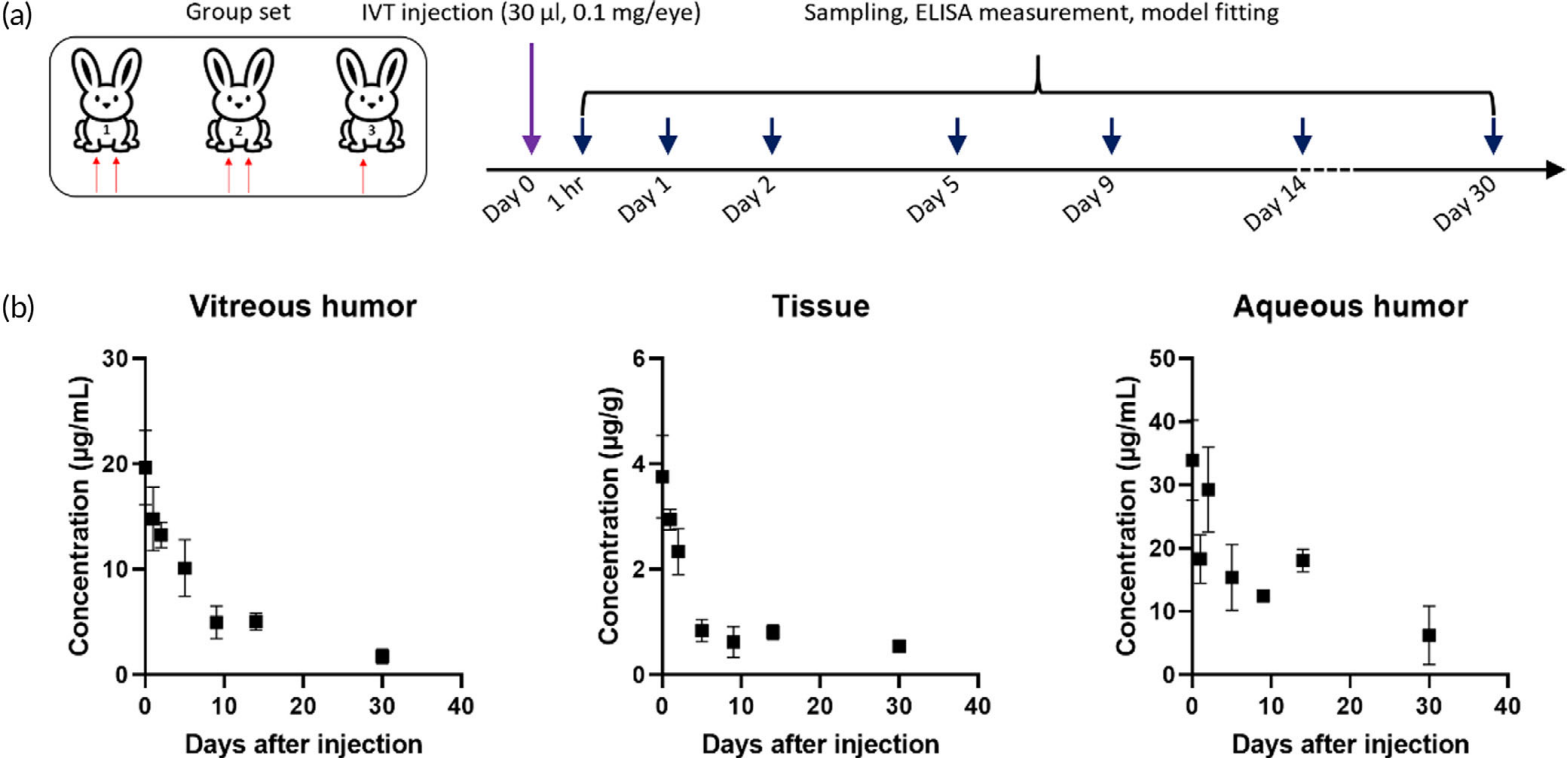
Pharmacokinetic analyses of nanoFc in the rabbit eye. (a) Schematic representation of the injection strategy and sampling pattern; (b) dependence of drug concentration on days after injection for nanoFc in the vitreous humor, tissue and aqueous humor, respectively.

In order to evaluate the ocular pharmacokinetics of our novel Fc‐fused multivalent nanobodies, we administered nanoFc to rabbit eyes and measured their concentration as a function of time after injection. Each time point was evaluated in three rabbits, in which five eyes were injected with drugs and one eye was injected with buffer (Figure 7a). This arrangement was used to assess the interference of drugs in two single eyes and ensure the independence of data acquired from two eyes in a rabbit. We found that the concentration of nanoFc in non‐injected eye was negligible compared to that in the injected eye (data not shown). Therefore, we considered the data acquired from each eye of a rabbit as independent and disregarded the interference of drug injected in individual eyes. During the experiment, we observed cataract in a rabbit treated with nanoFc. The drug concentration in the cataract eye was lower than that in the normal eye (data not shown), so we did not include this animal in our data analysis. One possible explanation for these findings could be inadvertent contact with the crystalline lens during injection, which could result in the injection of some drugs into the lens and subsequently cause traumatic cataracts. However, during analysis, the crystalline lens was removed, leading to a decrease in the overall concentration of the drug.

Figure 7b illustrates the dependence of ocular drug concentration on days after injection for nanoFc. With logarithmical processing, we found that vitreous PK profiles followed a first‐order elimination pathway. Therefore, we applied the corresponding models to fit the data and acquired PK parameters. In the vitreous humor, the half‐life *t*_1/2_ was determined to be 5.33 days, which is longer or comparable to the half‐life (4.60, 3.92, or 5.10 days) of aflibercept as measured elsewhere.^39^, ^40^, ^41^ Additionally, we noticed that the drug concentration at tissue remained as 25% of the initial concentration from day 5 to day 30, indicating limited elimination of the drug through the tissue. We also observed that aqueous drug concentrations from 1 h after injection to day 14 were high, suggesting that nanoFc was more likely to be eliminated through the aqueous pathway.

## 4 CONCLUSION

Despite significant efforts made over the last year to reduce the viscosity of highly‐concentrated antibodies, including formulation optimization, protein engineering, and protein suspensions,^24^, ^42^, ^43^ breakthrough outcomes have been limited. In this study, a novel molecule was developed that achieved an architectural‐level improvement, enabling high affinity, high concentration, and low viscosity. The nanoFc demonstrated high binding affinity, in vitro bioactivity, and satisfactory stability in high concentration formulation. Notably, the 148 mg/mL formulation surpasses the concentration of any proteins approved for the treatment of wAMD, enabling a higher molar dose upon injection and potentially prolonging the duration of anti‐VEGF action, reducing the frequency of intravitreal injections. The performance of the novel protein, nanoFc, is attributed to its unique molecule design, which improves affinity through multivalency rather than amino acid mutations. It eliminates “bad” amino acids and possesses a different architecture compared to conventional antibodies. In summary, this research proposes strategies for the development of high‐affinity and high concentration proteins for ocular delivery in wAMD treatment.

## AUTHOR CONTRIBUTIONS

**Chengnan Huang:** Conceptualization (equal); investigation (equal); methodology (equal); writing – original draft (equal). **Yuelin Wang:** Data curation (equal); investigation (equal); methodology (equal); project administration (equal). **Jinliang Huang:** Formal analysis (equal); methodology (equal); validation (equal). **Huiqin Liu:** Conceptualization (equal); investigation (equal). **Zhidong Chen:** Data curation (equal); software (equal). **Yang Jiang:** Formal analysis (equal); investigation (equal); resources (equal); writing – review and editing (equal). **Youxin Chen:** Conceptualization (equal); funding acquisition (equal); resources (equal); supervision (equal). **Feng Qian:** Conceptualization (equal); formal analysis (equal); funding acquisition (equal); resources (equal); supervision (equal); writing – review and editing (equal).

## CONFLICT OF INTEREST STATEMENT

The authors declare that no conflict of interests.

### PEER REVIEW

The peer review history for this article is available at https://www.webofscience.com/api/gateway/wos/peer‐review/10.1002/btm2.10632.

## Supporting information


**Data S1.** Supporting Information

## Data Availability

The data that support the findings of this study are available from the corresponding author upon reasonable request.

## References

[1] Ambati J , Fowler BJ . Mechanisms of age‐related macular degeneration. Neuron. 2012;75(1):26‐39. 22794258 10.1016/j.neuron.2012.06.018 PMC3404137

[2] Gottlieb JL . Age‐related macular degeneration. JAMA. 2002;288(18):2233‐2236. 12425683 10.1001/jama.288.18.2233

[3] Seah I , Zhao X , Lin Q , et al. Use of biomaterials for sustained delivery of anti‐VEGF to treat retinal diseases. Eye (Lond). 2020;34(8):1341‐1356. 32001821 10.1038/s41433-020-0770-y PMC7376230

[4] Regula JT , Lundh von Leithner P , Foxton R , et al. Targeting key angiogenic pathways with a bispecific CrossMAb optimized for neovascular eye diseases. EMBO Mol Med. 2016;8(11):1265‐1288. 27742718 10.15252/emmm.201505889 PMC5090659

[5] Yang S , Zhao J , Sun X . Resistance to anti‐VEGF therapy in neovascular age‐related macular degeneration: a comprehensive review. Drug Des Dev Ther. 2016;10:1857‐1867. 10.2147/DDDT.S97653 PMC4898027 27330279

[6] Basile AS , Hutmacher M , Nickens D , et al. Population pharmacokinetics of pegaptanib in patients with neovascular, age‐related macular degeneration. J Clin Pharmacol. 2012;52(8):1186‐1199. 21947371 10.1177/0091270011412961

[7] Ng EW , Shima DT , Calias P , Cunningham ET Jr , Guyer DR , Adamis AP . Pegaptanib, a targeted anti‐VEGF aptamer for ocular vascular disease. Nat Rev Drug Discov. 2006;5(2):123‐132. 16518379 10.1038/nrd1955

[8] Hageman GS , Gehrs K , Johnson LV , Anderson D . Age‐Related Macular Degeneration (AMD) . Webvision, viewed 5 June 2023. https://webvision.med.utah.edu/book/part‐xii‐cell‐biology‐of‐retinal‐degenerations/age‐related‐macular‐degeneration‐amd/

[9] Holz FG , Schmitz‐Valckenberg S , Fleckenstein M . Recent developments in the treatment of age‐related macular degeneration. J Clin Invest. 2014;124(4):1430‐1438. 24691477 10.1172/JCI71029 PMC3973093

[10] de Oliveira Dias JR , de Andrade GC , Novais EA , Farah ME , Rodrigues EB . Fusion proteins for treatment of retinal diseases: aflibercept, ziv‐aflibercept, and conbercept. Int J Retina Vitreous. 2016;2:3. 27847621 10.1186/s40942-016-0026-y PMC5088480

[11] Yannuzzi NA , Freund KB . Brolucizumab: evidence to date in the treatment of neovascular age‐related macular degeneration. Clin Ophthalmol. 2019;13:1323‐1329. 31413539 10.2147/OPTH.S184706 PMC6661993

[12] Muller S , Junker S , Wilke T , et al. Questionnaire for the assessment of adherence barriers of intravitreal therapy: the ABQ‐IVT. Int J Retina Vitreous. 2021;7(1):43. 34078475 10.1186/s40942-021-00311-x PMC8170736

[13] Jacobs B , Palmer N , Shetty T , et al. Patient preferences in retinal drug delivery. Sci Rep. 2021;11(1):18996. 34556761 10.1038/s41598-021-98568-7 PMC8460733

[14] Ehlken C , Ziemssen F , Eter N , et al. Systematic review: non‐adherence and non‐persistence in intravitreal treatment. Graefes Arch Clin Exp Ophthalmol. 2020;258(10):2077‐2090. 32572607 10.1007/s00417-020-04798-2 PMC7550304

[15] Hurand V , Ducloyer JB , Baudin F , et al. IMPACT study: impact of adherence to anti‐VEGF intravitreal injections for macular disease during COVID 19‐related confinement in France. Acta Ophthalmol. 2023;101(1):91‐99. 35765939 10.1111/aos.15206 PMC9350166

[16] Falavarjani KG , Nguyen QD . Adverse events and complications associated with intravitreal injection of anti‐VEGF agents: a review of literature. Eye (Lond). 2013;27(7):787‐794. 23722722 10.1038/eye.2013.107 PMC3709385

[17] Mehta SC , Kelley RF , Tesar DB . Protein conjugates and fusion proteins as ocular therapeutics. Drug Discov Today. 2019;24(8):1440‐1445. 31202674 10.1016/j.drudis.2019.05.025

[18] Li H , Lei N , Zhang M , Li Y , Xiao H , Hao X . Pharmacokinetics of a long‐lasting anti‐VEGF fusion protein in rabbit. Exp Eye Res. 2012;97(1):154‐159. 21933673 10.1016/j.exer.2011.09.002

[19] Stewart MW . The study of intravitreal drug pharmacokinetics: does it matter? And if so, how? Expert Opin Drug Metab Toxicol. 2018;14(1):5‐7. 29221430 10.1080/17425255.2018.1416098

[20] Veritti D , Sarao V , di Bin F , Lanzetta P . Pharmacokinetic and pharmacodynamic rationale for extending VEGF inhibition increasing intravitreal aflibercept dose. Pharmaceutics. 2023;15(5):1416. 37242658 10.3390/pharmaceutics15051416 PMC10220677

[21] Brown DM . Evaluation of 8 mg intravitreal aflibercept injection for neovascular age‐related macular degeneration: results from the phase 2 CANDELA study. Invest Ophthalmol Vis Sci. 2022;63(7):1345–F0179.

[22] Rabia LA , Desai AA , Jhajj HS , Tessier PM . Understanding and overcoming trade‐offs between antibody affinity, specificity, stability and solubility. Biochem Eng J. 2018;137:365‐374. 30666176 10.1016/j.bej.2018.06.003 PMC6338232

[23] Chardès V , Vergassola M , Walczak AM , Mora T . Affinity maturation for an optimal balance between long‐term immune coverage and short‐term resource constraints. Proc Natl Acad Sci U S A. 2022;119(8):e2113512119. 35177475 10.1073/pnas.2113512119 PMC8872716

[24] Wang S , Zhang N , Hu T , et al. Viscosity‐lowering effect of amino acids and salts on highly concentrated solutions of two IgG1 monoclonal antibodies. Mol Pharm. 2015;12(12):4478‐4487. 26528726 10.1021/acs.molpharmaceut.5b00643

[25] Huang C , Huang J , Zhu S , Tang T , Chen Y , Qian F . Multivalent nanobodies with rationally optimized linker and valency for intravitreal VEGF neutralization. Chem Eng Sci. 2023;270:118521.

[26] Andreas G , Rene GO , Joachim B , Marie‐Ange B , Erik D . Bispecific Binding Molecules Binding to VEGF and Ang2. US10414828B2. 2019.

[27] Wang L , Xu GL , Gao K , et al. Development of a robust reporter‐based assay for the bioactivity determination of anti‐VEGF therapeutic antibodies. J Pharm Biomed Anal. 2016;125:212‐218. 27042807 10.1016/j.jpba.2016.03.042

[28] Wang X , Bove AM , Simone G , Ma B . Molecular bases of VEGFR‐2‐mediated physiological function and pathological role. Front Cell Dev Biol. 2020;8:599281. 33304904 10.3389/fcell.2020.599281 PMC7701214

[29] Zhang Y , Huo M , Zhou J , Xie S . PKSolver: an add‐in program for pharmacokinetic and pharmacodynamic data analysis in Microsoft excel. Comput Methods Programs Biomed. 2010;99(3):306‐314. 20176408 10.1016/j.cmpb.2010.01.007

[30] Kanai S , Liu J , Patapoff TW , Shire SJ . Reversible self‐association of a concentrated monoclonal antibody solution mediated by fab‐fab interaction that impacts solution viscosity. J Pharm Sci. 2008;97(10):4219‐4227. 18240303 10.1002/jps.21322

[31] Czajkowsky DM , Hu J , Shao Z , Pleass RJ . Fc‐fusion proteins: new developments and future perspectives. EMBO Mol Med. 2012;4(10):1015‐1028. 22837174 10.1002/emmm.201201379 PMC3491832

[32] Beck A , Wagner‐Rousset E , Ayoub D , van Dorsselaer A , Sanglier‐Cianférani S . Characterization of therapeutic antibodies and related products. Anal Chem. 2013;85(2):715‐736. 23134362 10.1021/ac3032355

[33] Zbacnik TJ , Holcomb RE , Katayama DS , et al. Role of buffers in protein formulations. J Pharm Sci. 2017;106(3):713‐733. 27894967 10.1016/j.xphs.2016.11.014

[34] Kenneth SG , Saurabh W . High concentration vegf receptor fusion protein containing formulations. US20190343918A1. 2019.

[35] Sankar K , Krystek SR Jr , Carl SM , Day T , Maier JKX . AggScore: prediction of aggregation‐prone regions in proteins based on the distribution of surface patches. Proteins. 2018;86(11):1147‐1156. 30168197 10.1002/prot.25594

[36] Raut AS , Kalonia DS . Viscosity analysis of dual variable domain immunoglobulin protein solutions: role of size, electroviscous effect and protein‐protein interactions. Pharm Res. 2016;33(1):155‐166. 26286186 10.1007/s11095-015-1772-5

[37] Mehl JW , Oncley JL , Simha R . Viscosity and the shape of protein molecules. Science. 1940;92(2380):132‐133. 17730219 10.1126/science.92.2380.132

[38] Yang JT . The viscosity of macromolecules in relation to molecular conformation. Adv Protein Chem. 1961;16:323‐400. 14002447 10.1016/s0065-3233(08)60032-7

[39] Christoforidis JB , Williams MM , Kothandaraman S , Kumar K , Epitropoulos FJ , Knopp MV . Pharmacokinetic properties of intravitreal I‐124‐aflibercept in a rabbit model using PET/CT. Curr Eye Res. 2012;37(12):1171‐1174. 22991959 10.3109/02713683.2012.727521

[40] Park SJ , Choi Y , Na YM , et al. Intraocular pharmacokinetics of intravitreal aflibercept (Eylea) in a rabbit model. Invest Ophthalmol Vis Sci. 2016;57(6):2612‐2617. 27258433 10.1167/iovs.16-19204

[41] Joo K , Park SJ , Choi Y , et al. Role of the Fc region in the vitreous half‐life of anti‐VEGF drugs. Invest Ophthalmol Vis Sci. 2017;58(10):4261‐4267. 28850637 10.1167/iovs.17-21813

[42] Huang C , Chen L , Franzen L , Anderski J , Qian F . Spray‐dried monoclonal antibody suspension for high‐concentration and low‐viscosity subcutaneous injection. Mol Pharm. 2022;19(5):1505‐1514. 35417176 10.1021/acs.molpharmaceut.2c00039

[43] Geoghegan JC , Fleming R , Damschroder M , Bishop SM , Sathish HA , Esfandiary R . Mitigation of reversible self‐association and viscosity in a human IgG1 monoclonal antibody by rational, structure‐guided Fv engineering. MAbs. 2016;8(5):941‐950. 27050875 10.1080/19420862.2016.1171444 PMC4968137

