# Supplementary material for: A bioengineered anti‐VEGF protein with high affinity and high concentration for intravitreal treatment of wet age‐related macular degeneration
Source: Bioeng Transl Med. 2023 Dec 19;9(2):e10632. doi: 10.1002/btm2.10632 (PMC10905556; doi:10.1002/btm2.10632)
Supplement: Supplementary file 1 — Data S1. Supporting Information [file BTM2-9-e10632-s001.docx]

**A Bioengineered Anti-VEGF Protein with High Affinity and High Concentration for Intravitreal Treatment of Wet Age-Related Macular Degeneration**

**(Supporting materials)**

Chengnan Huang^1†^, Yuelin Wang^2,3^, Jinliang Huang^4^, Huiqin Liu^4^, Zhidong Chen^1^, Yang Jiang^2,3*^, Youxin Chen^2,3*^, Feng Qian^1*^

^1^ School of Pharmaceutical Sciences, Beijing Frontier Research Center for Biological Structure, and Key Laboratory of Bioorganic Phosphorus Chemistry & Chemical Biology (Ministry of Education), Tsinghua University, Beijing, 100084, P.R. China

^2^ Department of Ophthalmology, Peking Union Medical College Hospital, Peking Union Medical College, Chinese Academy of Medical Sciences, Beijing, 100730, P.R. China

^3^ Key Lab of Ocular Fundus Diseases, Chinese Academy of Medical Sciences, Beijing, 100730, P.R. China

^4^ Quaerite Biopharm Research, Beijing, 100195, P.R. China

*To whom correspondence should be addressed:

Dr. Yang Jiang, Email: [jiangyangpumch@126.com](mailto:jiangyangpumch@126.com)

Prof. Youxin Chen, Email: [chenyx@pumch.cn](mailto:chenyx@pumch.cn)

Prof. Feng Qian, Email: [qianfeng@tsinghua.edu.cn](mailto:qianfeng@tsinghua.edu.cn)

†Present address:

Department of Anesthesia, University of California at San Francisco, San Francisco, California 94110-1305.


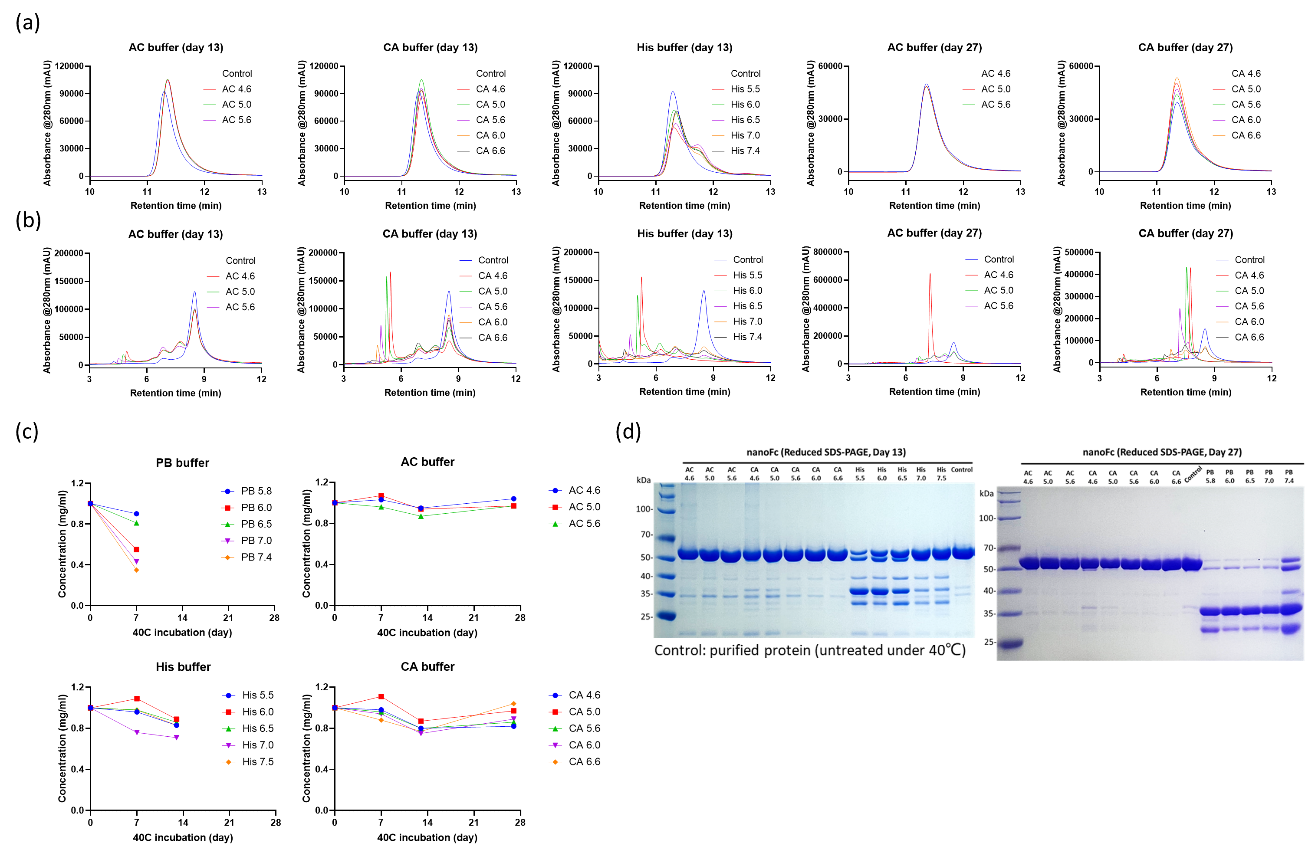
**Figure S1.** Effects of buffer systems (including buffer type and pH) on the stability of nanoFc. The experiment was conducted with a test concentration of 4 mg/mL and a test temperature of 40 ℃. (a) SEC-HPLC profiles; (b) WCX profiles; (c) Concentration; (d) SDS-PAGE. The following buffer systems were used: Sodium Dihydrogen Phosphate - Sodium Dihydrogen Phosphate (PB), Acetic Acid - Sodium Acetate (AC), Citric Acid - Sodium Citrate (CA), and Histidine - Histidine·HCl (His), with the number following the buffer name representing the pH value.


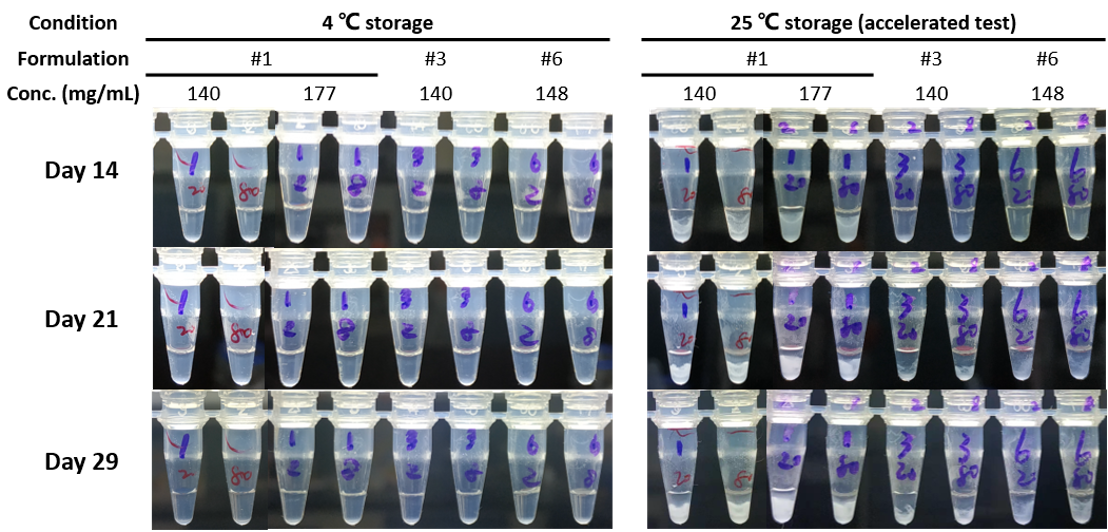
**Figure S2.** Appearance of highly-concentrated nanoFc in various formulations under conditions of 4 ℃ or 25 ℃. The information for each formulation is listed in **Table S2**. 2 or 20 on the tube represents Tween-20, while 8 or 80 represents Tween-80.


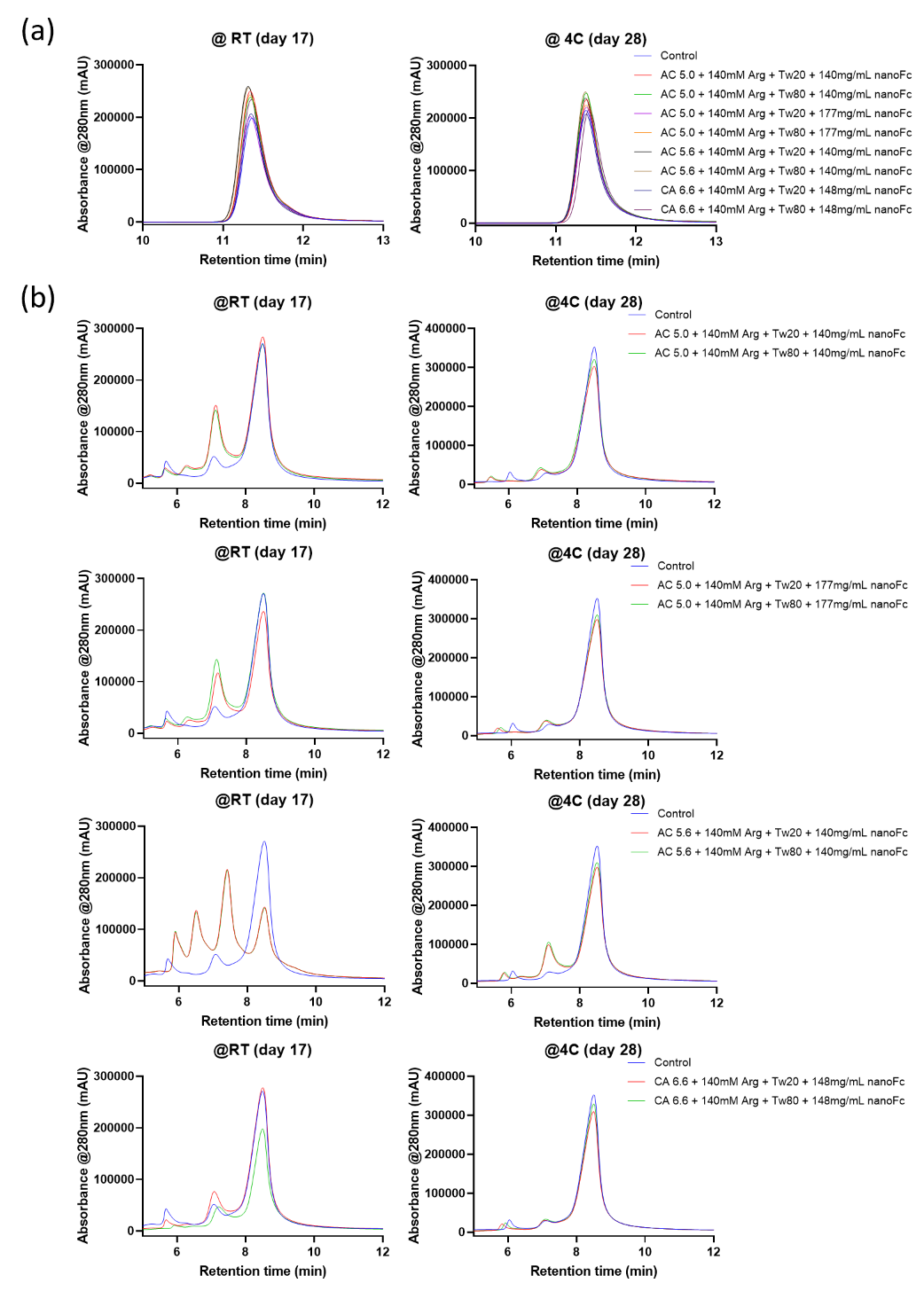


**Figure S3.** Stability characterization of highly-concentrated nanoFc in various formulations. (a) SEC-HPLC and (b) WCX profiles.

**Table S1.** Formulations considered for the development of a highly-concentrated nanoFc solution.

| **#** | **Buffers** | | **Salts or sugars** |
| --- | --- | --- | --- |
| **1** | 20 mM AC | pH 5.0 | 140 mM arginine·HCl |
| **2** |  | pH 5.0 | 140 mM NaCl |
| **3** |  | pH 5.6 | 140 mM arginine·HCl |
| **4** |  | pH 5.6 | 140 mM NaCl |
| **5** |  | pH 5.6 | 40 mM NaCl, 5% sucrose |
| **6** | 20 mM CA | pH 6.6 | 140 mM arginine·HCl |
| **7** |  | pH 6.6 | 140 mM NaCl |
| **8** |  | pH 6.6 | 40 mM NaCl, 5% sucrose |

**Table S2.** Selected formulations for the accelerated study of highly-concentrated nanoFc solutions.

| **#** | **Buffers** | **Salts or sugars** | **Surfactants** |
| --- | --- | --- | --- |
| **1** | 20 mM AC, pH 5.0 | 140 mM arginine·HCl | 0.02% Tween-80/20 |
| **3** | 20 mM AC, pH 5.6 | 140 mM arginine·HCl | 0.02% Tween-80/20 |
| **6** | 20 mM CA, pH 6.6 | 140 mM arginine·HCl | 0.02% Tween-80/20 |
